# Pulmonary aspiration of gastric contents in two patients taking semaglutide for weight loss

**DOI:** 10.1002/anr3.12278

**Published:** 2024-01-14

**Authors:** S. A. Avraham, J. Hossein, F. Somri, N. Hawash, O. Hochman

**Affiliations:** ^1^ Department of Anaesthesia Bnei Zion Medical Center, Rappaport Faculty of Medicine Technion‐Israel Institute of Technology Haifa Israel; ^2^ Department of Anaesthesia Bnei Zion Medical Center, Rappaport Faculty of Medicine Technion‐Israel Institute of Technology Haifa Israel; ^3^ Faculty of Medicine Università degli Studi “Gabriele d'Annunzio” Chieti Pescara Italy; ^4^ Department of Anaesthesia Bnei Zion Medical Center, Rappaport Faculty of Medicine Technion‐Israel Institute of Technology Haifa Israel; ^5^ Bnei Zion Medical Center, Rappaport Faculty of Medicine Technion‐Israel Institute of Technology Haifa Israel

**Keywords:** aspiration, gastric emptying, GLP‐1 agonist

## Abstract

Semaglutide is a new weight loss treatment that has received substantial media attention in recent years. Anaesthetists must be aware of a potentially dangerous side effect of the drug: decreased gastric emptying. This is caused by effects on gastric smooth muscle, mediated by the vagal afferent nerves. This is especially relevant in the peri‐operative setting where pulmonary aspiration of gastric contents is a recognised complication. Here, we report two cases of peri‐operative regurgitation of gastric contents in patients taking semaglutide. A patient taking semaglutide may have a full stomach despite compliance with routine pre‐operative fasting guidelines. We consider how to manage patients receiving glucagon‐like peptide‐1 agonist therapy in the peri‐operative period, including identifying those at high risk of regurgitation. Precautions such as rapid sequence induction and tracheal intubation can be used, but gastric ultrasound may also be useful in the pre‐operative environment to help identify patients at high risk of aspiration.

## Introduction

Glucagon‐like peptide (GLP)‐1 agonist therapy is a non‐insulin‐based approach that is combined with diet and exercise to treat type 2 diabetes mellitus and obesity. It has gained recognition as an effective weight loss treatment with only mild side effects. The most notable are nausea, vomiting and diarrhoea [1]. Here, we report two cases in which the use of GLP‐1 agonist therapy for weight loss may have resulted in delayed gastric emptying, with subsequent regurgitation and pulmonary aspiration of gastric contents.

## Report

### Case 1

A 70‐year‐old man with a body mass index (BMI) of 35 kg.m^−2^ and a medical history of ischaemic heart disease, type 2 diabetes mellitus, hypertension and chronic obstructive pulmonary disease presented for elective endoscopic retrograde cholangiopancreatography due to cholidocholelithiasis. His medications included spironolactone, aspirin, tamsulosin, metformin, empagliflozin, ramipril and semaglutide, taken as a 1 mg weekly subcutaneous injection. His last dose of semaglutide was 6 days prior to the procedure. Pre‐operative physical examination was unremarkable; he was orientated and alert, and had no nausea or vomiting. Blood glucose was 8.8 mmol.l^−1^ (HbA1C 7%). He had undergone surgical procedures in the past uneventfully. He gave informed consent to undergo surgery under general anaesthetic (GA).

The anaesthetist decided to perform rapid sequence induction and intubation, a decision made because of the patient's BMI and history of diabetes mellitus. Prior to induction, the patient had been nil by mouth for food and fluids for over 12 h. He had stable respiratory and haemodynamic parameters, with oxygen saturation of 95% on room air, blood pressure 132/72 mmHg and a heart rate of 105 beats.min^−1^.

The patient received pre‐oxygenation with 100% oxygen for 5 min, after which etomidate 20 mg and rocuronium 100 mg were given intravenously. During laryngoscopy, a large volume of regurgitated particulate gastric content including undigested food was observed. Laryngoscopy and tracheal intubation were performed using a standard laryngoscope with a size 4 Macintosh blade and an 8‐mm tracheal tube. Nasogastric suction was performed. Oxygen/air was delivered, with an F_I_O_2_ of 0.95. Bag ventilation was performed, initially requiring peak ventilatory pressures of up to 35 cm H_2_O. Despite this, the oxygen saturation deteriorated to 83%. After these measures were continued for 5 min, the situation improved. At this stage, anaesthesia was maintained with sevoflurane (1.1 minimum alveolar concentration, MAC), with a peak ventilatory pressure of 28 cm H_2_O; the oxygen saturation was 95%. A chest radiograph was ordered, showing bilateral infiltrates with fluid in the dependent segment of the right lower lobe. Because observations were stable, it was agreed that the procedure should go ahead, which was carried out successfully. The patient was then transferred to the intensive care unit and tracheal extubation was performed the next day. The patient was discharged after a week.

### Case 2

A 25‐year‐old woman with a BMI of 32 presented for incision and drainage of a breast abscess under general anaesthesia. The only medication she was taking was semaglutide 1 mg weekly subcutaneous injection, for weight loss. Her last dose of semaglutide was 4 days prior to surgery. Physical examination was unremarkable. She had received anaesthesia in the past without problems. She gave informed consent to undergo surgery under GA.

The patient had fasted from food and fluids for over 8 h. General anaesthesia was induced using 3 mg midazolam, 200 mcg fentanyl and 200 mg propofol. A size 4 laryngeal mask airway was used, and anaesthesia was maintained with sevoflurane (1.0 MAC). At the end of uneventful surgery, it was confirmed that the patient was breathing spontaneously with normal observations, and the supraglottic airway was removed. Following supraglottic airway removal, the patient began to cough and showed signs of respiratory distress. Regurgitation of solid and liquid stomach content was observed, and her oxygen saturation fell to 85%; her respiratory rate was observed to be 14. The patient's head was tilted to the right and the oral cavity was suctioned with a nasogastric tube. Because of the desaturation, we decided to re‐induce general anaesthesia with propofol 200 mg and succinylcholine 100 mg, and perform tracheal intubation using a standard laryngoscope with a size 3 Macintosh blade and a 7.5 mm tracheal tube. General anaesthesia was maintained with sevoflurane (1.0 MAC) for 20 min once respiratory parameters were stabilised. Sevoflurane was stopped, and tracheal extubation was performed once the patient was fully awake. Her heart rate was 90 beats.min^−1^, blood pressure was 135/88 mm Hg and oxygen saturation was 97%. In the post‐anaesthesia care unit, observations were normal, and the chest radiograph was unremarkable.

## Discussion

Semaglutide is a GLP‐1 receptor agonist that was first marketed over 15 years ago, initially designed for glycaemic control in diabetic patients. By mimicking the GLP‐1 hormone released by the gut in response to food, semaglutide increases endogenous insulin secretion to lower blood glucose. The 2021 STEP 2 trial demonstrated semaglutide to be an effective weight loss drug, reporting 10% weight loss among subjects [2]. It was approved by the US Food and Drug Administration to be used as a weight loss agent among some overweight and obese patients. Since 2021, semaglutide has gained recognition as a ‘celebrity weight‐loss jab’. There has been great demand for it that has led to worldwide shortage. It is currently available in three different forms (subcutaneous injection for children, subcutaneous injection for adults and tablet form). We are not the first group to suggest a connection between semaglutide and peri‐operative aspiration [3]. However, we hope to add to the existing literature and encourage future research that might lead to improved peri‐operative practice for patients taking semaglutide.

Semaglutide appears to delay gastric emptying by impeding antral and duodenal motility while at the same time stimulating pyloric tightening [4]. Inhibition of postprandial acid secretion also appears to contribute to this ‘ileal brake’ mechanism through direct effects on gastric smooth muscle [5]. The effect on gastric smooth muscle appears to be mediated largely by vagal afferent nerves [6]. Delayed gastric emptying is a known risk factor for pulmonary aspiration during anaesthesia [7]. Gudin et al. report a 3.5‐fold increase in the rate of peri‐operative pulmonary aspiration in patients using drugs such as GLP‐1 agonists [8].

It should be noted that both of our patients continued to use semaglutide in the week prior to anaesthesia. We think that GLP‐1 agonist therapy may have resulted in delayed gastric emptying and led to pulmonary aspiration of gastric contents. Although we are unable to prove causation, we agree with Klein and Hobai [3] that there is ‘significant cause for concern’, given the known mechanism of these drugs. Non‐compliance with the pre‐operative fasting regimen could have been the cause of peri‐operative aspiration. This seems unlikely in our cases because our patients were noted to be very engaged in safe preparation for their anaesthetic.

The American Society of Anesthesiologists (ASA) released pilot guidelines in June 2023 [9], addressing GLP‐1 receptor agonist therapy and their relationship to peri‐operative aspiration events. The recommendation for patients taking semaglutide is to withhold the medication for a week prior to anaesthesia, based on its 7‐day half‐life. If on the day of the procedure gastrointestinal symptoms such as severe nausea, vomiting, bloating or abdominal pain are present despite having discontinued the drug, consideration should be given to delaying the procedure. If the patient has no gastrointestinal symptoms but did not stop the medication, it is advised to either use ‘full stomach’ precautions or to use ultrasound guidance to evaluate stomach contents.

Pulmonary aspiration is one of the highest risk complications of airway management. According to the 2021 ASA Closed Claims analysis, 57% of aspiration incidents result in death, and another 15% result in permanent severe injury [10]. Bedside ultrasound appears to be emerging as an effective modality to aid in assessing gastric content, and future studies using gastric ultrasound may help to elucidate the effects of semaglutide and whether omitting for 7 days is sufficient. We hope our two cases help to raise awareness of this potential clinical problem with an increasingly common drug.

## References

[1] Frias JP , Davies MJ , Rosenstock J , et al. Tirzepatide versus semaglutide once weekly in patients with type 2 diabetes. New England Journal of Medicine 2021; 385: 503–515. 34170647 10.1056/NEJMoa2107519

[2] Davies M , Faerch L , Jeppesen OK , et al. Semaglutide 2·4 mg once a week in adults with overweight or obesity, and type 2 diabetes (STEP 2): a randomized, double‐blinded, double‐dummy, placebo‐controlled, phase 3 trial. Lancet 2021; 397: 971–984. 33667417 10.1016/S0140-6736(21)00213-0

[3] Klein SR , Hobai IA . Semaglutide, delayed gastric emptying, and intraoperative pulmonary aspiration: a case report. Canadian Journal of Anesthesia 2023; 70: 1394–1396. 36977934 10.1007/s12630-023-02440-3

[4] Schirra J , Wank U , Arnold R , Göke B , Katschinski M . Effects of glucagon‐like peptide‐1(7–36)amide on motility and sensation of the proximal stomach in humans. Gut 2002; 50: 341–348. 11839712 10.1136/gut.50.3.341 PMC1773139

[5] Perfetti R , Merkel P . Glucagon‐like Peptide‐1: a major regulator of pancreatic beta‐cell function. European Journal of Endocrinology 2000; 143: 717–725. 11124853 10.1530/eje.0.1430717

[6] Imeryuz N , Yegen BC , Bozkurt A , et al. Glucagon‐like peptide‐1 inhibits gastric emptying via vagal afferent‐mediated central mechanisms. American Journal of Physiology 2002; 273: 920–927. 10.1152/ajpgi.1997.273.4.G920 9357836

[7] Kallar SK , Everett LL . Potential risks and preventive measures for pulmonary aspiration: new concepts in preoperative fasting guidelines. Anesthesia and Analgesia 1993; 77: 171–182. 8100406 10.1213/00000539-199307000-00034

[8] Gudin B , Ladhari C , Robin P , et al. Incretin‐based drugs and intestinal obstruction: a pharmacovigilance study. Thérapie 2020; 75: 641–647. 32418731 10.1016/j.therap.2020.02.024

[9] American Society of Anesthesiologists . American Society of Anesthesiologists Consensus‐Based Guidance on Preoperative Management of Patients (Adults and Children) on Glucagon‐Like Peptide‐1 (GLP‐1) Receptor Agonists. 2023. https://www.asahq.org/about‐asa/newsroom/news‐releases/2023/06/american‐society‐of‐anesthesiologists‐consensus‐based‐guidance‐on‐preoperative.

[10] Warner MA , Meyerhoff KL , Warner ME , Posner KL , Stephens L , Domino KB . Pulmonary aspiration of gastric contents: a closed claims analysis. Anesthesiology 2021; 135: 284–291. 34019629 10.1097/ALN.0000000000003831

